# Implementation Strategies to Address Cardiometabolic Disparities in Black Men: Lessons from Existing Research and Future Directions

**DOI:** 10.1007/s11892-025-01597-z

**Published:** 2025-07-08

**Authors:** Jaclynn Hawkins

**Affiliations:** School of Social Work, Department of Learning Health Sciences (DLHS), Medical School, University of Michigan, 1080 S University Ave, Ann Arbor, MI 48109 USA

**Keywords:** Implementation science, Cardiometabolic health, Black men, Health disparities, CFIR, RE-AIM, FRAME

## Abstract

**Purpose:**

Black men in the United States experience a disproportionate burden of cardiometabolic diseases, including type 2 diabetes (T2DM), hypertension, and cardiovascular disease (CVD). Despite these disparities, existing interventions often fail to address the shared risk factors, structural determinants, and implementation barriers that impact engagement and sustainability. This review applies implementation science frameworks to evaluate strategies for improving cardiometabolic interventions tailored to Black men.

**Recent Findings:**

Community-based interventions, culturally tailored health education programs, and peer-led models have demonstrated success in improving cardiometabolic outcomes for Black men. However, challenges such as medical mistrust, underrepresentation in research, and systemic barriers continue to limit their reach and sustainability. The Consolidated Framework for Implementation Research (CFIR) identifies multi-level barriers and facilitators, the Reach, Effectiveness, Adoption, Implementation, and Maintenance (RE-AIM) framework assesses intervention impact, and the Framework for Reporting Adaptations and Modifications to Evidence-Based Interventions (FRAME) highlights equity-driven adaptations.

**Summary:**

Applying implementation science frameworks provides structured insights into optimizing interventions for Black men by addressing barriers across patient, provider, and system levels. Key facilitators include culturally relevant adaptations, an inclusive healthcare workforce, and trusted community partnerships. Future research should integrate equity-focused implementation strategies to improve adoption, engagement, and long-term sustainability of cardiometabolic interventions for Black men.

## Introduction

Cardiometabolic diseases, including type 2 diabetes (T2DM), hypertension, and cardiovascular disease (CVD), represent a significant public health challenge in the United States, affecting millions of adults and contributing to considerable morbidity and mortality [1–4]. These conditions frequently co-occur, compounding the risk of complications, hospitalizations, and premature mortality compared to their isolated occurrence [5–7]. Multimorbidity–the coexistence of two or more chronic conditions–has become the norm rather than the exception in cardiometabolic disease management, presenting critical challenges for intervention design, implementation, and sustainability [8–11]. 

Black men disproportionately experience a higher burden of cardiometabolic diseases, including uncontrolled hypertension, T2DM and chronic kidney disease, which substantially increase their risk of early mortality and complicate disease management [12–17]. Despite these glaring disparities, research and interventions have often been rooted in single-disease models that fail to address the shared risk factors, social determinants, and interconnected mechanisms underlying cardiometabolic conditions [18–20]. This fragmented approach underestimates the collective impact of these diseases and limits the scalability and effectiveness of interventions for Black men. Addressing these disparities requires a shift toward integrated, multi-level strategies that account for structural, provider-level, and individual-level barriers.

Systemic racism, socioeconomic disadvantage, medical mistrust, limited access to healthcare, and underrepresentation in clinical trials drive persistent inequities in the prevention, diagnosis, and management of cardiometabolic diseases among Black men [21–23]. Compounding these challenges, many interventions are not designed with Black men in mind, resulting in low adoption, limited engagement, and poor long-term sustainability. Addressing these disparities requires a nuanced understanding of the implementation barriers and facilitators specific to this population, as well as strategies to equitably adapt and scale evidence-based practices (EBPs). Notably, few EBPs have been intentionally developed for Black men; instead, most involve adaptations of interventions originally designed for the general population—underscoring a critical gap in tailored intervention development.

This review synthesizes existing research, identifies key gaps, and offers actionable recommendations to improve the effectiveness and long-term sustainability of cardiometabolic health interventions for Black men. It centers on evaluating implementation strategies for community-based and system-level interventions, given their potential to generate broader impact and lasting change. In light of persistent disparities in cardiometabolic outcomes among Black men, it is critical to explore how implementation science has been applied within this context. While the existing evidence base remains limited, this review not only consolidates current findings but also draws attention to areas in need of further study—laying the groundwork for more equitable and scalable approaches. Insights from adjacent fields such as chronic disease management, HIV prevention, and cancer care inform this analysis and highlight opportunities to improve outcomes through multi-level, equity-focused strategies. Ultimately, this work aims to advance the use of implementation science to design sustainable, community-driven solutions that reflect and respond to the lived experiences of Black men.

### Theoretical Framework

Implementation science frameworks offer structured approaches for understanding and addressing these complex challenges. This review retrospectively applies implementation science frameworks to cardiometabolic interventions to assess (1) implementation processes across individual, provider, and system levels and (2) barriers and facilitators to implementation. Three frameworks were chosen based on their distinct yet complementary contributions to understanding implementation processes: these frameworks are among the most commonly utilized in health services research and collectively provide comprehensive coverage of implementation domains relevant to cardiometabolic interventions for Black men. These frameworks are the Consolidated Framework for Implementation Research (CFIR), the Reach, Effectiveness, Adoption, Implementation, and Maintenance (RE-AIM) framework, and the Framework for Reporting Adaptations and Modifications to Evidence-Based Interventions (FRAME). CFIR identifies barriers and facilitators to adoption, considering intervention characteristics, organizational factors, and community engagement. By tailoring strategies to specific healthcare settings, CFIR improves implementation success. RE-AIM evaluates implementation across five dimensions: reach (the absolute number, proportion, and representativeness of individuals who participate), effectiveness (impact on targeted outcomes, including positive and negative effects, quality of life, and economic outcomes), adoption (organizational uptake), implementation (fidelity to the intervention’s protocol, including consistency of delivery and time and cost of implementation), and maintenance (the extent to which an intervention becomes part of routine practice at the setting level, AND the long-term effects on outcomes at the individual level). It helps researchers assess both intervention effectiveness and real-world feasibility, particularly for Black men. Finally, FRAME ensures interventions are equitably adapted while maintaining fidelity, preventing modifications from compromising core components. Together, these frameworks provide a strong foundation for evaluating and optimizing cardiometabolic interventions across diverse healthcare and community settings [24–28].

## Methods

A narrative review approach was used to identify lessons learned from existing research and inform future directions by retrospectively applying implementation science frameworks to evaluate barriers, facilitators, development, and adaptations in cardiometabolic interventions. Given the limited number of available studies, narrative analysis was appropriate because it allows for a qualitative synthesis of diverse findings, highlighting why certain implementation strategies succeed or fail and how contextual factors shape intervention outcomes. This approach enables a more nuanced understanding of emerging themes and gaps in the literature, which may not be captured through more rigid systematic review methods [28, 29].

A comprehensive search was conducted across four electronic databases: PubMed, Scopus, Web of Science, and Google Scholar. The search focused on peer-reviewed journal articles published between 2015 and 2025 to ensure the inclusion of recent advancements in implementation science and cardiometabolic health interventions. Search terms included a combination of Medical Subject Headings (MeSH) and keywords related to cardiometabolic diseases (e.g., “type 2 diabetes,” “hypertension,” “cardiovascular disease”), implementation strategies (e.g., “health interventions,” “RE-AIM,” “CFIR,” “FRAME”), health disparities (e.g., “racial disparities,” “Black men,” “health equity”), and community-based approaches (e.g., “peer-led programs,” “barbershop-based interventions,” “faith-based health interventions”). Studies were included if they met the following criteria: (1) examined implementation processes for health-focused interventions targeting Black men; (2) focused on community-based, provider-level, or system-level interventions; (3) reported on barriers, facilitators, or adaptations relevant to improving engagement and outcomes for Black men; and (4) were published in English in a peer-reviewed journal. Studies were excluded if they did not include race-specific data, concentrated exclusively on clinical effectiveness without addressing contextual factors, or lacked relevance to implementation processes.

A standardized data extraction template captured key study characteristics, including author, publication year, setting, population, intervention type, and implementation strategies (see Table 1). This systematic approach ensured consistent evaluation of implementation processes across studies and facilitated the application of implementation science frameworks to identified barriers and facilitators. To evaluate implementation strategies, the three selected implementation science frameworks provided distinct yet complementary perspectives. The CFIR framework helped contextualize implementation challenges by examining structural inequities, organizational readiness, and other multi-level influences. RE-AIM supported the assessment of intervention impact by analyzing how well programs engaged Black men, achieved intended outcomes, and sustained long-term reach. FRAME highlighted the role of culturally responsive adaptations in ensuring interventions remained both equitable and effective without compromising fidelity. This integrated approach provided a comprehensive lens through which to evaluate the complex implementation landscape for cardiometabolic interventions targeting Black men.

An integrative thematic synthesis grouped studies by implementation processes and mapped findings to frameworks to identify recurring themes and gaps. CFIR examined multi-level barriers and facilitators, RE-AIM assessed reach, adoption, fidelity, and sustainability, and FRAME evaluated culturally relevant adaptations and equity considerations. To mitigate limitations of retrospective framework application, data extraction and mapping were conducted systematically, with careful documentation of decisions and justifications. Missing data were noted, and conservative assumptions were applied when aligning findings with framework constructs. Context preservation was prioritized, particularly regarding cultural and community factors and the historical context of health disparities, ensuring that findings accurately reflected real-world implementation challenges. Also, to ensure rigor and transparency, this review followed the Standards for Reporting Implementation Studies (StaRI) guidelines [29], facilitating the identification of research gaps and equity-focused recommendations for improving cardiometabolic interventions for Black men.

**Table 1:** Data extraction template for cardiometabolic interventions targeting black men

Column	Description	Example
Author/Year	Citation information for the study	Victor et al. (2011)
Study Design	Methodology used	Cluster-randomized controlled trial
Population	Demographic characteristics of participants	Black men ages 35–65 with uncontrolled hypertension
Setting	Environment where intervention was implemented	Community barbershops in urban areas
Intervention Type	Primary approach used	Peer-led hypertension management program
Implementation Strategies	Methods used to enhance implementation	Barber training, clinical pharmacy integration, community partnership
Barriers Identified	Challenges to implementation	Medical mistrust, transportation limitations, work schedule conflicts
Facilitators Identified	Factors enhancing implementation	Trusted community setting, culturally tailored materials, peer influence
Framework Mapping	Relevant constructs from implementation frameworks	CFIR: Outer setting (community partnerships), Inner setting (readiness) RE-AIM: Reach (engagement), Adoption (setting) FRAME: Context adaptations (barbershop setting)

## Results

### Implementation Processes Across Individual, Provider, and System Levels

The research on implementation strategies addressing cardiometabolic disparities among Black men remains limited, particularly strategies targeting patients, healthcare providers, and healthcare systems [20, 21]. However, existing approaches–such as culturally tailored health education programs, peer-led models, barbershop-based interventions, and policy-level changes–offer valuable insights into enhancing engagement, adoption, and sustainability of evidence-based interventions while also addressing the unique barriers faced by Black men [21, 30–33].

### Patient-Level Strategies

Patient-level strategies prioritize culturally tailored interventions that improve health literacy, support self-management behaviors, and enhance treatment adherence by addressing the specific needs and barriers faced by Black men. Through application of the CFIR framework, these strategies can be examined by considering how they incorporate culturally relevant intervention characteristics, leverage trusted community networks to address external influences (outer settings), and enhance individual capacity through education and support. This approach allows for systematic assessment of both the strategies themselves and the contextual factors that influence their implementation. Community-based interventions, such as peer-led barbershop programs, exemplify this approach by creating culturally appropriate environments that promote health management. For example, Victor et al. (2011) demonstrated the effectiveness of barbershop-based interventions in managing hypertension, leveraging trusted community spaces to foster engagement and address outer setting factors such as trust and cultural norms [34]. Griffith et al. (2013) highlighted how male peer influence motivates Black men to adopt healthier behaviors, illustrating the role of peer models in enhancing individual-level factors such as self-efficacy and motivation [30]. Similarly, Luque et al. (2014) further emphasized how characteristics of interventions, in this case barbershop-based programs, can empower community leaders to deliver culturally relevant health education, making health messaging more impactful and increasing intervention effectiveness [35]. While these strategies have shown promise, scalability and long-term sustainability remain significant challenges due to resource demands. This underscores the need for systemic investment and infrastructure to support their broader implementation.

### Provider-Level Strategies

Provider-focused strategies aim to improve workforce diversity, deliver implicit bias training, and incorporate peer models to foster culturally competent care. These strategies fall within CFIR’s inner setting (e.g., organizational readiness and training) and individual characteristics (e.g., healthcare providers’ skills and attitudes) domains. Of note, increasing representation among Black healthcare providers has been shown to enhance trust and patient engagement. Promising interventions for enhancing cultural competence among providers include structured cultural humility training programs, interactive case-based learning, and community immersion experiences. For example, programs that combine didactic education with experiential learning in community settings have demonstrated significant improvements in providers’ understanding of cultural factors influencing health behaviors among Black men. Additionally, ongoing mentorship by culturally competent senior providers and involvement of community members as educators have shown positive impacts on care delivery and patient satisfaction.

For instance, Powell et al. (2019) reported that medical mistrust and perceived racism in healthcare can delay preventive health screenings among Black men, emphasizing the need for culturally tailored care and diverse healthcare teams [31]. Adams et al. (2017) documented that medical mistrust is a significant barrier to colorectal cancer screening among African Americans, underscoring the need for culturally tailored approaches to improve engagement and screening uptake [32]. Rayford et al. (2021) further underscored the importance of informed, diverse care teams in addressing the unique genomic and immunological differences in prostate cancer among Black men [33], Lillard et al. (2022) examined the drivers of prostate cancer disparities among Black men, including medical mistrust and poor physician-patient communication, and identified clinician education and patient empowerment as central to more equitable care [36]. Despite their potential, these strategies face systemic barriers, including underrepresentation of Black providers in both direct-service and leadership roles in health care systems and insufficient resources for implicit bias training.

### System-Level Strategies

Cardiometabolic interventions targeting system-level strategies address structural determinants of health through policy reforms and community-based interventions. These strategies can be characterized by CFIR’s outer setting (e.g., external policies and community needs) and inner setting (e.g., organizational readiness and resources). Of note, Barbershop-based interventions illustrate a community-based model that integrates health education and screenings into trusted local spaces. For example, Linnan et al. (2014) synthesized the literature on health promotion programs delivered in barbershops and beauty salons, showing how these trusted community settings can reach diverse populations and help address health disparities [37]. Such interventions can be adapted to create culturally appropriate environments that address barriers to participation and improve access to tailored health education [37–39].

At the policy level, Medicaid expansion under the Affordable Care Act (ACA) has addressed financial barriers to care, leading to higher rates of diabetes and hypertension screenings among low-income Black adults [40]. This expansion facilitated timely diagnosis and treatment of chronic conditions for underserved populations, addressing structural determinants of health at the policy level [40]. Despite these advances, challenges related to sustainability and integration persist, as these programs often rely on external funding and lack system-wide adoption.

### Bridging Levels of Implementation

Barbershop interventions exemplify strategies that bridge patient-level and system-level implementation. At the patient level, they provide culturally tailored health education and empower individuals to manage their health. At the system level, they build infrastructure within trusted community spaces and establish pathways to improve access to care through collaborative models. This dual impact highlights the versatility and effectiveness of community-based interventions in addressing the complex health disparities experienced by Black men.

### Barriers and Facilitators to Implementation

**Fig. 1:**
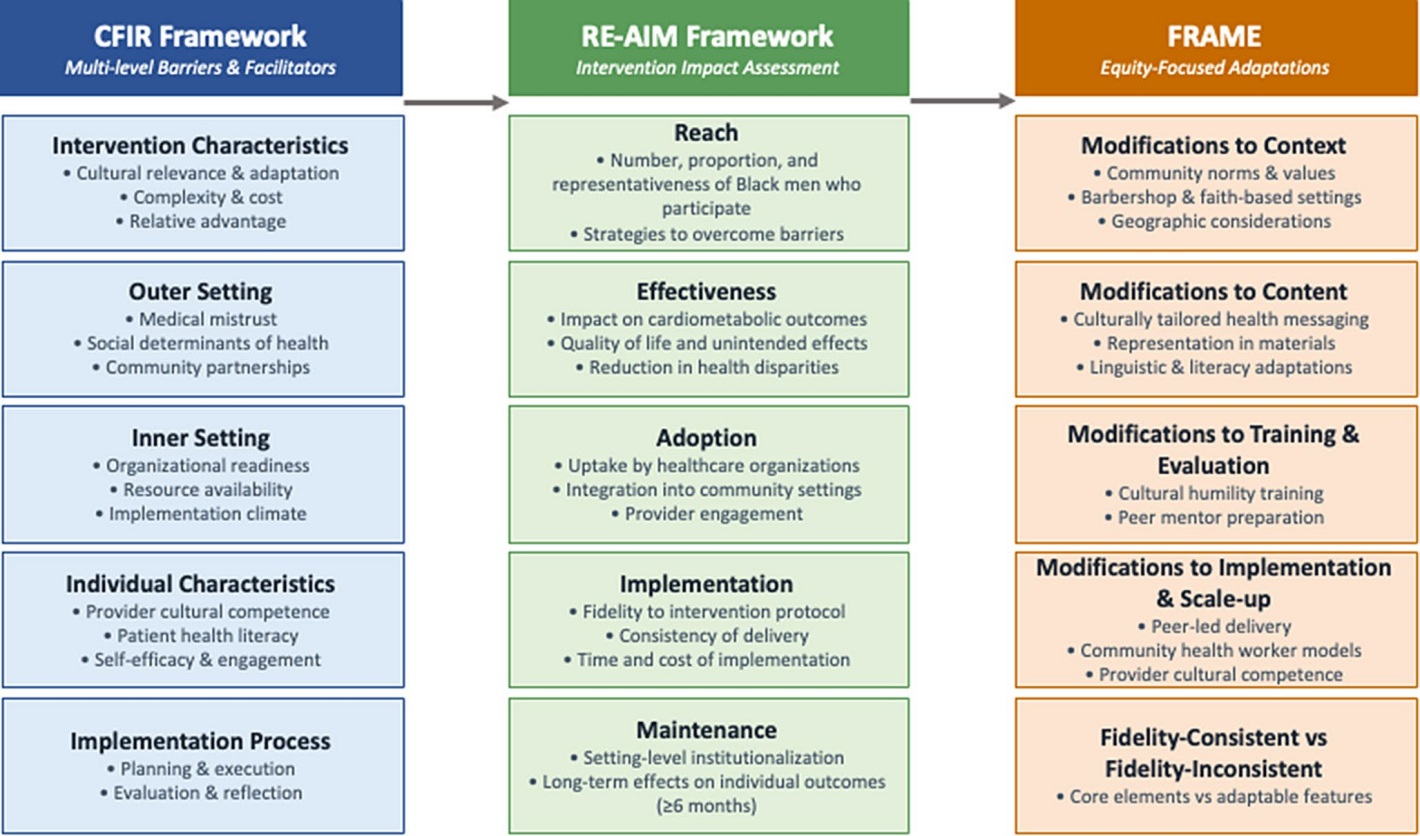
Application of Implementation Science Frameworks to Address Cardiometabolic Disparities in Black Men. Note: Showing the integrated application of CFIR, RE-AIM, and FRAME frameworks with arrows connecting specific components between frameworks

The success of cardiometabolic interventions for Black men depends on navigating a complex array of barriers and facilitators across patient, provider, and system levels. Application of CFIR, RE-AIM, and FRAME, provide structured approaches to analyze and address these challenges. Figure 1 illustrates how these frameworks interact to inform the identification and mitigation of implementation barriers while enhancing facilitators at multiple levels. This integrated approach allows for comprehensive assessment of how interventions can be optimized for Black men. CFIR identifies barriers and facilitators within key domains, such as the outer setting (e.g., structural inequities, community factors) and inner setting (e.g., organizational culture, resource availability) [23]. RE-AIM evaluates how these factors influence intervention reach, effectiveness, and sustainability, while FRAME provides insights into the equity-driven adaptations needed to overcome obstacles and enhance cultural relevance.

### Barriers to Implementation

#### Medical Mistrust

Medical mistrust remains one of the most pervasive barriers to engaging Black men in cardiometabolic interventions [30, 31, 41–44]. This mistrust is deeply rooted in historical injustices, such as the Tuskegee Syphilis Study, and persists due to ongoing racial disparities in healthcare access and outcomes [40, 45]. Within CFIR, this barrier falls under the outer setting domain, reflecting how societal and historical factors shape perceptions of the healthcare system. For example, Alsan et al. (2019) demonstrated that Black men were significantly more likely to engage in preventive health visits when treated by Black providers, highlighting the potential to mitigate mistrust through increased representation and culturally concordant care [42].

Using RE-AIM, mistrust affects the reach and adoption of interventions, as programs may fail to engage participants if they do not address underlying concerns or involve trusted community leaders. Community-led research partnerships have shown promise in reducing medical mistrust and improving engagement. For example, the Men of Color Health Awareness (MOCHA) program utilized a community-based participatory research approach where community members served as equal partners in research design, implementation, and evaluation [46, 47]. This approach resulted in significantly higher participation rates and improved trust in health information compared to traditional researcher-led interventions. Similarly, the Partnership for Robust Minority Engagement in Clinical Trials (PROMPT) demonstrated that when Black men were involved in all stages of clinical trial development, from question formulation to data interpretation, both enrollment and retention rates improved substantially [48, 49]. FRAME underscores the importance of equity-driven adaptations, such as incorporating Black-led businesses, organizations and racially-congruent peers to foster trust and credibility. These approaches build relationships that reduce mistrust and improve participation.

### Limited Representation in Research and Clinical Trials

The underrepresentation of Black men in clinical trials and implementation research is a persistent barrier that limits the development of effective, culturally relevant interventions. For instance, fewer than 10% of participants enrolled in clinical trials are Black men, making it difficult to tailor interventions that adequately address their specific health needs [21]. This lack of representation means that many cardiometabolic interventions fail to consider social determinants of health—such as food insecurity, transportation barriers, and economic instability—that disproportionately impact Black men. CFIR categorizes this barrier within both intervention characteristics (e.g., lack of cultural tailoring) and the outer setting (e.g., misalignment with community needs). Analysis with the RE-AIM framework reveals that limited representation hinders the reach and effectiveness of interventions, as strategies developed without input from Black men may fail to resonate with their lived experiences. FRAME can be used to highlight missed opportunities to adapt interventions by incorporating culturally relevant components, such as community-specific dietary advice or addressing systemic barriers to accessing care.

Efforts to address this gap must prioritize targeted recruitment initiatives and the inclusion of Black men in intervention design to ensure that research findings translate into meaningful health improvements. Expanding community partnerships and integrating trusted messengers–such as barbers, and other peer models–into clinical research can enhance recruitment and retention while improving health outcomes.

### Facilitators to Implementation

#### Community Engagement and Trust-Building

Community-based interventions, particularly those conducted in culturally relevant settings such as barbershops and churches, have been shown to significantly improve health outcomes for Black men. Interventions based in trusted community spaces facilitate recruitment and retention by embedding health promotion efforts within familiar, culturally significant environments [21]. For example, barbershops provide an ideal setting for delivering health education and screenings because they offer a culturally congruent space where Black men feel comfortable discussing health-related topics [30]. Building on this, a study demonstrated the success of a intervention that trained barbers as lay health advocates, resulting in significant reductions in systolic blood pressure among participants [50].

Importantly, research indicates that effective peer models may differ across generations. For younger Black men (18–35), peer mentors with shared lived experiences and similar social contexts have proven most effective, with digital engagement strategies enhancing connection. Middle-aged men (36–55) respond well to respected community leaders and those who have successfully managed health challenges. For older adults (56+), healthcare partners from faith-based organizations and veterans’ groups have shown the strongest impact on engagement and behavior change. These generational differences highlight the importance of age-appropriate tailoring when developing peer-led interventions to ensure cultural and contextual relevance across the lifespan.

CFIR’s outer setting domain captures the importance of leveraging community norms and values to foster engagement, while FRAME assists in the identification of key adaptations such as training community leaders to deliver culturally relevant health messaging. Applying the RE-AIM framework, these interventions demonstrate substantial reach and adoption, though their long-term Maintenance often depends on securing institutional support and resources.

### Culturally Tailored Messaging and Delivery

Culturally tailored interventions are a key facilitator in addressing the specific needs, values, and preferences of Black men. These interventions integrate culturally relevant content, such as dietary guidance rooted in traditional foods, and employ communication strategies that resonate with the lived experiences of participants [30]. Research indicates that interventions lacking cultural saliency—those that do not align with the values and preferences of the target community—produce less effective results, leading to challenges with recruitment, retention, and engagement. For example, a diabetes prevention program that incorporated culturally specific dietary advice and motivational interviewing resulted in improved weight loss and reduced T2DM incidence among Black men [30]. Recent work emphasizes the importance of deep structure tailoring, which ensures that interventions address not only surface-level cultural elements (e.g., language, recruitment materials) but also deeper cultural values, lived experiences, and the social contexts that shape health behaviors [21]. CFIR identifies culturally tailored interventions as addressing intervention characteristics, making them more acceptable to participants. FRAME facilitates the identification of equity-driven adaptations, such as tailoring communication styles and incorporating community-specific knowledge, to ensure interventions are both effective and relevant.

### Use of Peer-Led Models

Peer-led models have proven particularly effective in engaging Black men in cardiometabolic interventions. Peer mentors serve as trusted liaisons between patients and healthcare systems, addressing social determinants of health, providing emotional support, and promoting care coordination [22, 30–32, 50]. Research suggests that Black men prefer receiving health information from peers rather than from traditional healthcare sources, reinforcing the effectiveness of peer-led interventions. For instance, Griffith et al. (2013) highlight how peer influence increases motivation for engaging in health behaviors such as physical activity [51]. Their study found that Black men were more likely to engage in exercise when encouraged by peers, who provided accountability, encouragement, and a shared cultural perspective. Similarly, a hypertension management program utilizing peer mentors reported improved blood pressure control and medication adherence among participants due to the culturally sensitive guidance peers provided [52].

Successful implementation of peer-led models requires comprehensive infrastructure and support. This includes structured training programs for peer mentors (typically 20–40 h covering health education, communication skills, and resource navigation), ongoing supervision and support from healthcare professionals, regular community of practice meetings to address challenges, and appropriate compensation that values peers’ time and expertise. Additionally, effective programs establish clear role definitions, integrate peers into clinical workflows, provide access to relevant patient information while maintaining privacy, and create formal referral pathways to connect patients with needed services. CFIR’s inner setting domain highlights the organizational readiness, training, and structural support required for successfully integrating peers. FRAME helps to illustrate how peer roles are adapted to reflect shared lived experiences and community ties, ensuring cultural alignment and enhancing trust.

## Discussion

This review highlights critical gaps and opportunities in implementing cardiometabolic health interventions for Black men. The interplay between community engagement, healthcare system capacity, and structural barriers shapes intervention effectiveness. While community-based approaches, provider-level strategies, and culturally tailored health education address key challenges such as medical mistrust and limited access to care, persistent systemic barriers–including structural racism and social determinants of health–continue to hinder intervention reach, adoption, and sustainability [31, 42].

Consistent with prior research, this review finds that culturally tailored, community-based interventions can significantly improve health outcomes for Black men. For example, barbershop-based health programs, as demonstrated by Victor et al. (2018), successfully reduced systolic blood pressure by embedding screenings and health education within trusted community spaces [50]. These programs leverage CFIR’s outer setting domain by engaging existing community networks. However, scalability and sustainability remain challenges, as many rely on external funding and volunteer labor. Addressing these limitations requires systemic investment and healthcare partnerships to ensure long-term viability [30, 39, 40].

Provider-focused strategies—including workforce diversity, implicit bias training, and peer-led support models—promote culturally competent care. These approaches align with CFIR’s inner setting and individual characteristics domains by fostering organizational readiness and addressing the unique needs of Black men. Research shows that Black patients experience better engagement and adherence when cared for by culturally competent providers [31–33]. Several training programs have demonstrated effectiveness in improving provider competence, including the Cultural Competence Training for Healthcare Providers (CCT-HP) program, which showed significant improvements in patient satisfaction and clinical outcomes through its multi-modal approach combining didactic sessions, simulated patient encounters, and community immersion experiences. Similarly, the Patient-Centered Cultural Sensitivity Training (PC-CST) program improved provider knowledge, attitudes, and behaviors through its focus on power-sharing and recognition of patients’ expertise regarding their own health experiences. However, these strategies face systemic barriers such as the underrepresentation of Black providers in leadership roles and limited institutional support for implicit bias training. While peer-driven models show promise, they require ongoing investment to sustain impact and adapt to evolving community needs. Expanding these strategies necessitates structural changes within healthcare organizations, including policy reforms and resource allocation.

Medical mistrust, rooted in historical and ongoing inequities, emerged as a recurring barrier. Black patients demonstrate greater trust and engagement with Black providers, underscoring the potential of workforce diversity in improving health outcomes [42]. However, systemic barriers in medical education and hiring perpetuate the underrepresentation of Black healthcare professionals, limiting the scalability of these interventions. While workforce diversity is often cited as a strategy for reducing disparities, further research is needed to clarify its mechanisms of impact and the conditions under which it is most effective for Black men.

Implicit bias training alone has shown limited effectiveness in producing long-term behavioral change or reducing structural inequities. Cultural humility training, which emphasizes ongoing self-reflection, power-sharing with patients, and respect for lay expertise, offers a more impactful approach [53]. By fostering continuous learning and prioritizing collaboration, cultural humility training can strengthen patient-provider relationships and address systemic barriers to equitable care. These approaches align with CFIR’s emphasis on community engagement and organizational readiness. However, sustained efforts are needed to ensure meaningful structural change and equitable representation within healthcare systems.

Social determinants of health—including economic stability, housing, education, and neighborhood disinvestment—disproportionately affect Black men, exacerbating health disparities [54]. Racial discrimination and societal stressors further contribute to chronic illnesses such as hypertension and mental health challenges [55, 56]. Gender norms also shape health behaviors and outcomes, highlighting the need for interventions that consider sociocultural influences [57]. Segregation-driven disparities in physical activity and smoking further illustrate the role of place-based factors in shaping health risks [58, 59]. Community-based interventions that engage trusted leaders and incorporate culturally relevant strategies can help mitigate these barriers by fostering trust and aligning health promotion efforts with community needs [60, 61]. However, sustained investment and systemic change remain essential for long-term success.

Although existing research provides valuable insights, the limited application of implementation science frameworks—including CFIR, RE-AIM, and FRAME—restricts the ability of studies to systematically address barriers and promote equity-driven adaptations. CFIR informs community engagement and organizational readiness, while RE-AIM evaluates intervention reach and effectiveness. FRAME ensures culturally and contextually appropriate adaptations. Together, these frameworks offer a structured approach to developing multi-level, equity-focused strategies that integrate community partnerships, promote workforce diversity, and align interventions with the lived experiences of Black men. Researchers can begin incorporating these frameworks by: (1) using CFIR during intervention planning to systematically identify potential barriers and facilitators across multiple levels; (2) designing data collection instruments that capture RE-AIM dimensions from the outset, including reach metrics that assess representation of Black men, effectiveness measures sensitive to cultural contexts, adoption indicators at organizational levels, implementation processes focused on fidelity and adaptation, and maintenance strategies for long-term sustainability; and (3) employing FRAME to document adaptation decisions and their rationale, ensuring modifications enhance rather than compromise intervention effectiveness for Black men. Early engagement with implementation experts and community stakeholders can facilitate appropriate framework application throughout the research process.

## Conclusion

This review identifies persistent gaps in the implementation of cardiometabolic health interventions for Black men, particularly in addressing systemic barriers such as structural racism and social determinants of health. The underrepresentation of Black men in clinical trials and the inconsistent application of implementation science frameworks further limit the development and scalability of culturally relevant interventions. By retrospectively applying CFIR, RE-AIM, and FRAME, this review synthesizes existing findings and highlights opportunities for equity-driven adaptations to enhance intervention sustainability and effectiveness.

A key strength of this review is its systematic application of implementation science frameworks, which provide a structured approach to evaluating multi-level barriers and facilitators. However, as a narrative review, it may have missed relevant studies, particularly those not explicitly focused on implementation processes. Additionally, the scarcity of research on implementation barriers and facilitators for cardiometabolic interventions targeting Black men constrains the ability to identify best practices. Much of the existing literature prioritizes clinical effectiveness over implementation strategies, limiting insights into optimizing adoption, reach, and sustainability. Furthermore, while the retrospective application of implementation science frameworks offers valuable insights, it may not fully capture the complexity of implementation challenges, as these frameworks were not originally integrated into study designs. These limitations reflect broader gaps in research rather than weaknesses of this review itself. By identifying these gaps, this review provides a foundation for future studies that systematically test, refine, and scale effective implementation strategies.

Future research must prioritize equity-driven adaptations, targeted recruitment strategies, and deeper community engagement to enhance the reach, adoption, and sustainability of evidence-based interventions. Implementation science frameworks such as CFIR, RE-AIM, and FRAME offer comprehensive roadmaps for designing and scaling interventions that address systemic barriers while promoting equity. To mitigate the disproportionate burden of cardiometabolic conditions among Black men, multi-level strategies should integrate community partnerships and align interventions with the lived experiences of this population. Achieving health equity requires sustained structural reforms and resource allocation to dismantle systemic inequities. Advancing these efforts will enable healthcare systems, policymakers, and researchers to make meaningful progress in reducing cardiometabolic health disparities and improving long-term outcomes for Black men.

## Key References

1. Adams LB, Richmond J, Corbie-Smith G, Powell W. Medical mistrust and colorectal cancer screening among African Americans. J Community Health. 2017;42(5):1044–61.
  Important -- This recent study illuminates crucial barriers to preventive screenings among Black men, highlighting the intersection of medical mistrust, stigma, and health literacy challenges that directly impact cardiometabolic health outcomes.
2. Lillard JW Jr, Moses KA, Mahal BA, George DJ. Racial disparities in Black men with prostate cancer: a literature review. Cancer. 2022;128(21):3787–95.
  Important -- This review synthesizes the drivers of prostate cancer disparities among Black men, including medical mistrust, poor physician-patient communication, and unequal access, and points to clinician education and patient empowerment as levers for more equitable care, lessons that transfer to cardiometabolic interventions.
3. Linnan LA, D'Angelo H, Harrington CB. A literature synthesis of health promotion research in salons and barbershops. Am J Prev Med. 2014;47(1):77–85.
  Important -- This synthesis reviews health promotion programs delivered in barbershops and beauty salons, trusted community settings that reach diverse and underserved populations, and distills lessons on using these venues to advance health equity, directly transferable to cardiometabolic outreach for Black men.
4. Victor RG, Ravenell JE, Freeman A, et al. Effectiveness of a barber-based intervention for improving hypertension control in black men: the BARBER-1 study: a cluster randomized trial. Arch Intern Med. 2011;171(4):342–50.
  Important -- This recent study specifically evaluates lifestyle interventions for cardiometabolic disease prevention in Black communities, offering practical insights into implementation challenges and facilitators in real-world settings.

## Data Availability

No datasets were generated or analysed during the current study.
