# Correction: Implementation Strategies to Address Cardiometabolic Disparities in Black Men: Lessons from Existing Research and Future Directions

**DOI:** 10.1007/s11892-026-01630-9

**Published:** 2026-05-22

**Authors:** Jaclynn Hawkins

**Affiliations:** School of Social Work, Department of Learning Health Sciences (DLHS), Medical School, University of Michigan, 1080 S University Ave, Ann Arbor, MI 48109 USA

**Correction: Current Diabetes Reports (2025) 25:41**

10.1007/s11892-025-01597-z

Two distinct correction notices are drafted below:

First, the published version of this article contains errors in the numbering of in-text citations and one mangled citation range that did not appear in the accepted manuscript. These errors were introduced during production and have been corrected.

Second, the original version of this article contained errors in the naming of one of the implementation science frameworks applied throughout the review and in the description of the dimensions of a second framework.

The original article has been corrected.

